# Increased intake of vegetables, but not fruits, may be associated with reduced risk of hip fracture: A meta-analysis

**DOI:** 10.1038/srep19783

**Published:** 2016-01-25

**Authors:** Si yang Luo, Yan Li, Hong Luo, Xin hai Yin, Du ren Lin, Ke Zhao, Guang lei Huang, Ju kun Song

**Affiliations:** 1 Department of Oral and Maxillary Surgery, Gui Zhou provincial people’s hospital, Guiyang, China

## Abstract

Association between dietary intake of vegetables and fruits and risk of hip fracture has been reported for many years. However, the findings remain inconclusive. We conducted a meta-analysis to evaluate the relationship between intake of vegetables and fruits, and risk of hip fracture. Literature search for relevant studies was performed on PubMed and Embase databases. Five observational studies were included in the meta-analysis. Summary hazard ratio (HR) with corresponding 95% confidence interval (CI) was calculated from pooled data using the random-effects model irrespective of heterogeneity. Sensitivity and subgroup analysis were performed to explore possible reasons for heterogeneity. The summary HR for hip fracture in relation to high intake vs. low intake of only vegetables, only fruits, and combined intake of fruits and vegetables, was 0.75 (95% CI, 0.61–0.92), 0.87 (95% CI, 0.74–1.04), and 0.79 (95% CI, 0.61–1.03), respectively. Subgroup analyses based on study design, geographical location, number of cases, and gender showed similar results. Increased intake of vegetables, but not fruits, was found to be associated with a lower risk of hip fracture. Large prospective clinical trials with robust methodology are required to confirm our findings.

Osteoporosis and osteoporotic fractures account for a considerable proportion of disability, morbidity and mortality worldwide, especially in the elderly population, which imposes a significant social and economic burden^1^,^2^. Of all fractures, the hip fracture is a major and growing public health problem, especially among the elderly. An estimated 1.6 million hip fractures occurred worldwide in the year 2000^3^. The number of people sustaining a hip fracture is expected to rise to 6.3 million by the year 2050 because of an increasing aging population^4^. Understanding the risk factors for and the underlying mechanisms of hip fracture would help to develop preventive strategies against osteoporotic fracture.

The etiopathogenesis of hip fracture is complex^5^. Osteoporosis, one of the major risk factors for hip fracture, is characterized by low bone mass and deterioration of bone tissue. A growing body of evidence implicates lifestyle factors as being among the key determinants of the development of osteoporosis^6^. Among the various lifestyle factors, diet is considered a critical modifiable factor that affects bone health^7^. Available evidence from cross sectional studies suggests a correlation of high dietary intake of vegetables and fruits with increased bone mineral density (BMD)^8^,^9^, and with lower longitudinal BMD loss in men^10^ and premenopausal women^11^. Results from observational studies that examined the association between dietary intake of vegetables and fruits and risk of hip fracture, have often been contradictory. While some studies have suggested an inverse association between vegetables and fruits intake and incidence of hip fractures^12^,^13^,^14^ and reduced risk of hip fracture only among elderly Chinese men but not women^15^, others found no evidence of such an association^16^,^17^ and, or even a positive association^18^. We conducted a meta-analysis to confirm the hypothesis that higher intake of vegetables and fruits is associated with decreased risk of hip fracture.

## Results

### Literature search and study characteristics and quality

**Figure 1:**
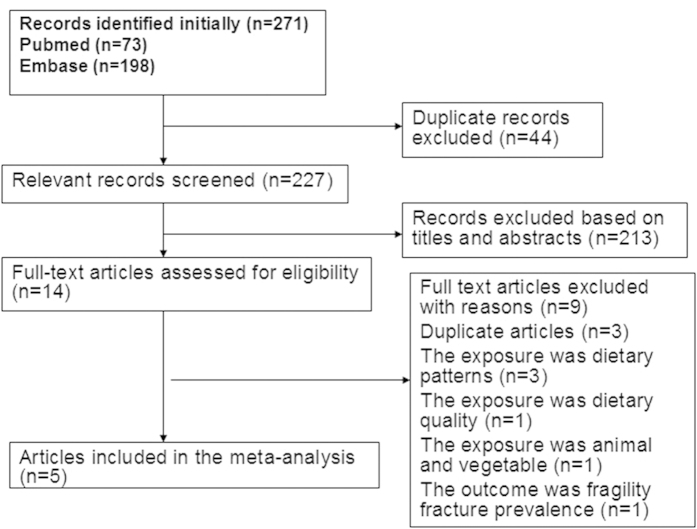
Schematic illustration of the literature search.

Figure 1 shows the flow diagram of the literature search. On initial search, 271 records were retrieved. After excluding the duplicate publications (N = 44), titles and abstracts of the remaining 227 articles were screened, and 213 articles excluded. The remaining 14 articles were full-text reviewed for further assessment. We further excluded three articles^19^,^20^,^21^ due to duplicate publications from the same study population. Three studies were excluded because they evaluated the association between dietary patterns and risk of hip fracture^17^,^22^,^23^. One study was excluded because it reported on the association between diet quality and risk of hip fracture^24^. Another study was excluded as it assessed the association between combined intake of animal and vegetable diet and hip fracture risk^25^. One study was excluded because it reported the association of dietary intake of deep-colored vegetables with the prevalence of osteoporosis fracture in postmenopausal Taiwanese women^16^. Finally, five articles qualified the criteria for inclusion in the meta-analysis (Fig. 1).

The descriptive data for all included studies are summarized in Tables 1, 2. The five studies included one case-control study^13^ and four cohort studies^12^,^14^,^15^,^18^, with a combined subject population of 330,417 participants and a total of 6,779 hip fractures came within the purview of our meta-analysis. Of the five studies, 3 were from Europe^12^,^14^,^18^ and 2 from China^13^,^15^. All studies separately reported data by intake of fruits or vegetables^12^,^13^,^14^,^15^,^18^, while in two studies the combined intake of fruit and vegetable intake was examined^12^,^13^. The mean duration of follow-up ranged from 4 to 14.2 years. In all the studies, sub-group analyses adjusted for conventional risk factors for hip fracture, such as age, gender, alcohol intake, total energy intake, physical activity and smoking, were reported.

As shown in the Table 2, the quality scores ranged from 6 to 8 (mean: 7.2).

### Combined intake of fruits and vegetables and the risk of hip fracture

**Figure 2:**
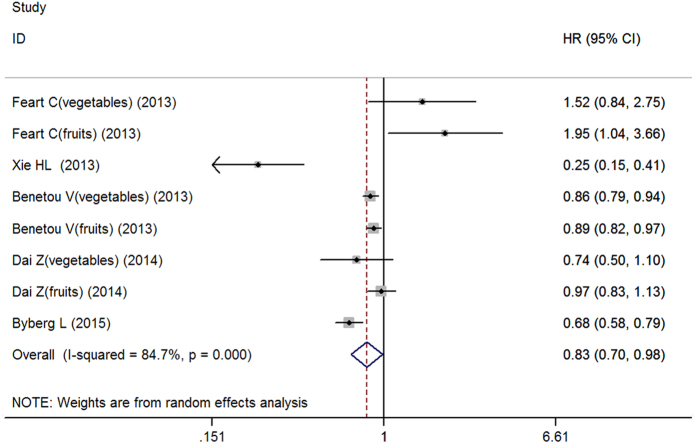
Forest plot for the association between total intake of vegetables plus fruits and hip fracture risk.

Eight studies from five articles were included in the analysis of combined intake of fruits and vegetables and the risk hip fracture. Pooled results indicated no association between combined intake of fruits and vegetables and the risk of hip fracture (OR: 0.83, 95% CI, 0.70–0.98; *P* = 0.028). However, significant heterogeneity was observed between the studies (*I*^2^ = 84.7%, heterogeneity *P* = 0.000) (Fig. 2). Sensitivity analysis showed unstable results, which ranged from 0.76 (95% CI, 0.65–0.90) with significant heterogeneity (*I*^2^ = 85.9%, heterogeneity *P* = 0.000) (excluding the study by Feart *et al*.^18^) to 0.88 (95%CI, 0.78–1.01) with significant heterogeneity (*I*^2^ = 73.5%, heterogeneity *P* = 0.001) (excluding the study by Xie *et al*.^13^). On subgroup analyses stratified by geographic location, study design, gender and number of cases, similar results were revealed (Table 3). The power of 63.23% was determined to detect an HR of 0.83 for the highest combined intake of fruits and vegetables intake compared with the lowest combined intake of fruits and vegetables intake.

### Intake of fruits and the risk of hip fracture

**Figure 3:**
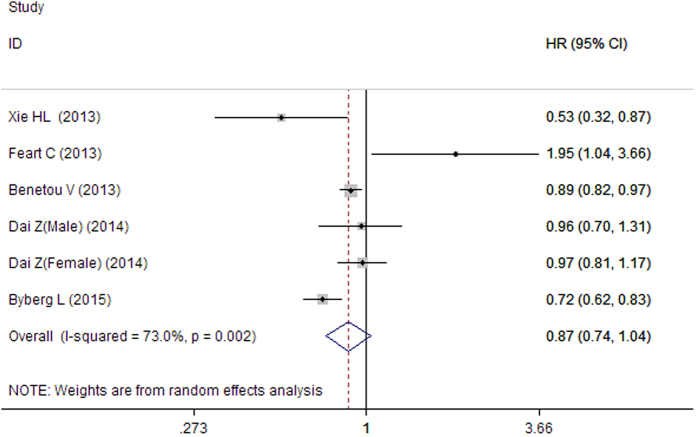
Forest plot for the association between fruit intake and hip fracture risk.

Six studies from five articles investigated the association between intake of fruits and risk hip fracture. The summary HR for highest versus lowest levels was 0.87 (95% CI: 0.74–1.04; *P* = 0.119) using the random-effects model, with a high heterogeneity (*I*^2^ = 73.0%, *P* = 0.002, Fig. 3). In sensitivity analysis, the unstable results was observed, which ranged from 0.84 (95%CI: 0.72–0.97) with notable heterogeneity (*I*^2^ = 66.8%, heterogeneity P = 0.017) (excluding the study by Feart *et al*.^18^) to 0.91 (0.77–1.07) with significant heterogeneity (*I*^2^ = 73.0%, heterogeneity P = 0.005) (excluding the study by Xie *et al*.^13^). Further, the subgroup analysis was also performed (Table 3). The power of 83.48% was determined to detect an HR of 0.87 for highest versus lowest category of total fruits intake.

### Intake of vegetables and the risk of hip fracture

**Figure 4:**
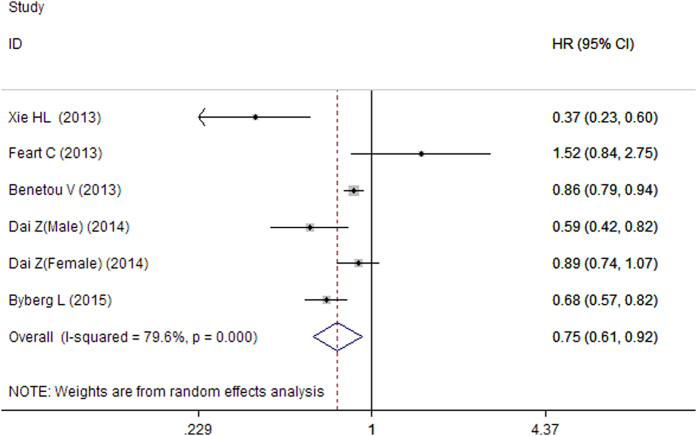
Forest plot for the association between intake of vegetables and hip fracture risk.

Six studies from five articles examined the association between intake of vegetables and the risk of hip fracture. The summary HR for highest versus lowest levels was 0.75 (95% CI: 0.61–0.92; *P* = 0.005) using a random-effects model, with a high heterogeneity (*I*^2^ = 79.6; *P* = 0.000, Fig. 4). Sensitivity analysis showed that the overall pooled estimate did not alter substantially with the exclusion of any one study. The overall combined OR after sequential exclusion of one study at a time ranged from 0.70 (95% CI, 0.57–0.86) with significant heterogeneity (*I*^2^ = 80.1, heterogeneity P = 0.000) (excluding the study by Feart *et al*.^18^) to 0.80 (95% CI, 0.68–0.96) with significant heterogeneity (*I*^2^ = 71.1, heterogeneity P = 0.008) (excluding the study by Xie *et al*.^13^). Further, the subgroup analysis was also performed (Table 3). The power of 98.34% was determined to detect an HR of 0.75 for highest versus lowest category of total vegetables intake.

## Discussion

Clinical practice recommends a daily intake of at least 5 servings of fruits and vegetables^26^. A higher intake of fruits and vegetables has been shown to reduce the risk of type 2 diabetes^27^, prolong life^28^, increase BMD^8^,^9^, decrease cardiovascular risk^29^, and risk of cancer^30^,^31^,^32^. Recently, evidence from multiple observational studies has shown the association between intake of fruits and vegetables and risk of hip fracture^12^,^13^,^14^,^15^,^16^,^17^,^18^. However, these studies have had a modest sample size and the strength of the association was variable among these studies, with HR varying from 0.06 (95% CI: 0.01–0.38) to 1.95 (95% CI, 1.04–3.66), with results across the studies being inconsistent. In case-control studies, higher consumption of fruits and vegetables was shown to be associated with a reduced risk of hip fracture among Chinese men and women^13^, and lower forearm fracture among postmenopausal women^33^. Adherence to a Mediterranean diet, rich in fruits and vegetables was found to be associated with lower risk of hip fracture in a cohort study^18^; while another cohort study suggested a higher risk of hip fracture^14^. At present, it is still unclear whether higher intake of fruits and vegetables is associated with lower risk of hip fracture. Therefore, we conducted a comprehensive meta-analysis to assess the association between the intake of fruits and vegetables and the risk of hip fracture. The present meta-analysis found that increased intake of vegetables, but not fruits, was inversely associated with the risk of hip fracture. Inferences drawn from pooled analysis tend to be more reliable than that from a single study as the overall HR is based on a large sample size and has an adequate statistical power. The power of the estimate of combined intake of vegetables and fruits, fruits, and vegetables with risk of hip fracture was 63.23%, 83.48% and 98.43%, respectively. Subgroup and sensitivity analyses revealed similar results, which serves to further strengthen the inferences drawn.

Several biological mechanisms may underlie the inverse association found in the meta-analysis. The fruits and vegetables are abundant in alkaline ions (potassium, magnesium and calcium). Calcium is a key element required to maintain bone health. The association of potassium and magnesium with bone health is well-documented^34^,^35^. The high vitamin K content in fruits and vegetables may help explain their role in maintaining bone health^36^. Further, fruits and vegetables are rich source of antioxidants (e.g., vitamin C, carotene and carotenoids), which by countering age-related oxidative stress and inflammation, may increase osteoclastogenesis and osteoclastic differentiation or suppress osteoblastic differentiation^37^. Furthermore, diets high in fruits and vegetables intake have lower dietary acid load^38^, which is known to inhibit osteoblast function and increase osteoclast activity resulting in reduced bone formation and increased bone resorption^39^. Finally, other nutrients such as polyphenols, tocopherols, tocotrienols and glutathione, may potentially contribute in reducing the risk of hip fracture^40^. However, evidence from animal and experimental studies suggests that reactive oxygen species (ROS), primarily derived from fruits and vegetables, may increase osteoclastogenesis^41^, and osteoclastic differentiation^42^, or inhibit osteoblastic differentiation^43^. Therefore, increased oxidative stress may play an important role in age-related bone loss that leads to osteoporotic fractures^44^. The preventive effect derived from the fruits and vegetables intake may counteract the adverse effect of ROS in turnover.

To the best of our knowledge this meta-analysis is the first to investigate the association between intake of fruits and vegetables and the risk of hip fracture. The large sample size and a longer time span serve to enhance the statistical power to detect possible associations. The power of HR value of intake of only fruits, and only vegetables with risk of hip fracture was 83.48% and 98.43%. Moreover, the use of multivariable-adjusted risk estimates minimized the confounding factors.

However, the potential limitations of this meta-analysis ought to be taken into account while interpreting the results. First, we cannot exclude the possibility that the observed inverse association between intake of vegetables and hip fracture risk could be owe to unmeasured or residual confounding. Further, though the meta-analysis involved a large number of participants, none of the included studies were interventional studies. Third, although we chose the highest multivariable adjusted effect estimates in our meta-analysis, we cannot rule out the effect of uncontrolled confounding by imprecise quantification of the dietary intake of fruits and vegetables. Fourth, a significant heterogeneity was detected among the studies. We further performed sensitivity and subgroup analyses to determine the sources of heterogeneity. The observed heterogeneity could partly be explained by study design (case-control studies and cohort studies) and the article^18^ reported the positive association. The statistical power of this French study (only included 57 incident hip fracture) was considerable lower, the positive association in the small number of cohort study could be observed by chance, which could be explained the high heterogeneity. In addition, the high heterogeneity observed in the case-control studies may be due in part to the high likelihood of recall and selection or memory bias and to the differential misclassification of exposure. Furthermore, unstable results were observed in subgroup and sensitivity analyses, which indicated that more relevant articles are needed to further explore this association. Fifth, the diet assessment methods and exposure ranges were different across the included studies, which may have contributed to the heterogeneity. Sixth, because the number of included studies were less than 10, we could not examine the publication bias, the influence of the results should not be ignored.

In summary, our results showed that intake of vegetables, but not fruits, may be associated with a lower risk of hip fracture. However, prospective studies need to be conducted to confirm the findings of this meta-analysis.

## Materials and Methods

### Literature search

A comprehensive literature search for relevant studies published up to May 2015 was performed in PubMed and EMBASE databases. The following search terms were used without imposing any limitation: “fruit(s)” or “vegetable(s)” or “brassicaceae” or “cruciferae” or “citrous” or “citrus”, combined with “hip fracture(s)”. The reference lists in the retrieved publications were manually screened to increase the yield of relevant studies.

### Study selection

Studies were considered eligible for inclusion if they met the following criteria: 1) study design: either cohort, case control or cross-sectional; 2) the exposure variable was fruit and/or vegetable consumption; 3) study outcomes included hip fracture risk; 4) Hazard ratio (HR)/Relative risk (RR) and Odds ratio (OR) (95% Confidence interval (CI) reported (or data to calculate these were available). The exclusion criteria were editorial letters, historical reviews and descriptive studies, such as case reports and case series, or laboratory studies. When multiple publications covered the same study population, only the study with the larger sample size was included. Two authors (JKS and HXY) independently performed the literature search and reviewed all studies for their eligibility for inclusion in the meta-analysis. Any disagreements were resolved by consensus or after consultation with the third author (GLH).

### Data extraction and assessment of methodological quality

Data pertaining to the following variables were independently extracted for each study by two authors (JKS and HXY) using a standardized data extraction form: first author’s surname, publication year, study design, country, number of subjects (cases/participants), gender, exposure type, methodology for dietary assessment, and covariates included in the multivariable model. Disagreements were resolved by discussion and a consensus reached by involving the third author (GLH), if necessary.

The methodological quality of the included studies was evaluated using the Newcastle-Ottawa scale (NOS)^45^, which contained 9 items for case-control and cohort studies, with every item having a score of 1 point. Studies with a total score of >6 were deemed to be of high quality.

### Statistical analysis

Hazard ratio with 95% CI was the common measure across all eligible studies. Since hip fracture is a relatively rare event, the differences among risk estimates (HR, RR and OR) were ignored, and the OR and RR were directly converted into HR. A random-effects model of DerSimonian and Laird was used to calculate summary HR, comparing the highest across all included studies regardless of heterogeneity^46^, which incorporates both intra- and inter-study variability. If a study did not report the total HR for combined intake of vegetables and fruits, but instead reported the HR for only vegetables and only fruits separately in a study, they were considered separately as if obtained from different studies, and used the effect estimate for that study in the meta-analysis of combined vegetables and fruits intake and hip fracture risk. If gender-specific estimates were available, they were also regarded as two different studies. Power analysis was performed using the method described by Cafri *et al*.^47^.

Sensitivity analysis was performed to evaluate robustness and stability of the results by sequentially omitting one study on each turn. Moreover, subgroup analysis was performed to assess heterogeneity and assess the influence of different inclusion criteria on the overall estimate.

## Additional Information

**How to cite this article**: Luo, S. *et al*. Increased intake of vegetables, but not fruits, may be associated with reduced risk of hip fracture: A meta-analysis. *Sci. Rep*. **6**, 19783; doi: 10.1038/srep19783 (2016).

## Figures and Tables

**Table 1:** Characteristic of studies included in the meta-analysis.

Study	Year	Population	Research	Study design	No. of patients	No. of subjects	Sex	Age,Median (Range),yrs	Dietary assessment	Duration(years)	Outcome ascertainment	Study period
Benetou V	2013	Europeans	EPIC study	A prospective cohort study	802	188795	W and M	48.6 (38.6–58.6)	Questionnaire	9	Telephone interviews, mailed questionnaire eliciting self-reported information, record linkage with hospital discharge records, hip and radius fracture registries	1992–2000
Feart C	2013	French	Three city study (3 C)	A prospective population cohort study	57	1482	W and M	75.9 (67.7–94.9)	FFQ	8	Self-reported history of fractures standardized 24-h dietary recall interview and dietary questionnaire	2001–2010
Xie HL	2013	Chinese	NA	A 1:1 matched case-control study	646	1292	W and M	70.8 (63.9–77.7)	FFQ-78	4	X-ray within 2 weeks of diagnosis	2008–2012
Dai Z	2014	Chinese	Singapore Chinese health study	A prospective population cohort study	1630	63257	W and M	NA (45–74)	FFQ-165	9.9	Surgical records or mecical records in the hospital discharge database of the Medicine System	1993–1998
Byberg L	2015	Swedish	Cohort of Swedish Men (COSM) and Swedish Mammography Cohort (SMC)	A prospective population cohort study	3644	75591	W and M	NA (45–83)	FFQ-14	14.2	Record linkage with national patient register	1998–2010

NA, not available; M, male; W, female.

**Table 2:**
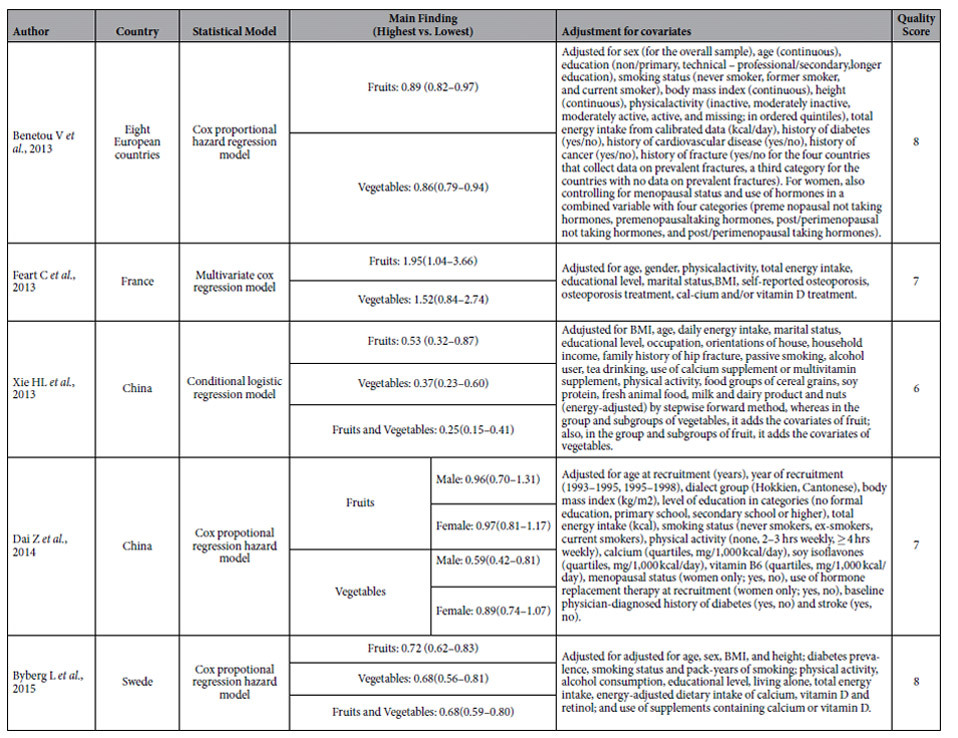
The Quality of Included Articles (n = 5).

**Table 3:** Results of subgroup analysis of total fruits, total vegetables, and both of them.

Subgroup analysis	Fruits				Vegetables				Both			
	n	HR(95%CI)	I^2^	P_for heterogeneity_	n	HR(95%CI)	I^2^	P_for heterogeneity_	n	HR(95%CI)	I^2^	P_for heterogeneity_
All studies	6	0.87(0.74–1.04)	73.0	0.002	6	0.75(0.61–0.92)	79.6	0.000	8	0.83(0.70–0.98)	84.7	0.000
Design												
Cohort	5	0.91(0.77–1.07)	73.0	0.005	5	0.81(0.68–0.96)	71.1	0.008	7	0.88(0.78–1.01)	73.5	0.001
Case-control	1	0.53(0.32–0.87)	NA	NA	1	0.37(0.23–0.60)	NA	NA	1	0.25(0.15–0.41)	NA	NA
Geograohical location												
European	3	0.90(0.69–1.17)	84.4	0.002	3	0.85(0.65–1.10)	78.1	0.010	5	0.89(0.75–1.04)	79.8	0.001
Asian	3	0.85(0.64–1.14)	60.5	0.080	3	0.60(0.37–0.97)	85.4	0.001	3	0.58(0.29–1.11)	92.5	0.000
No. of cases												
>100	5	0.84(0.72–0.97)	66.8	0.017	5	0.70(0.58–0.86)	80.1	0.000	6	0.76(0.65–0.90)	85.9	0.000
<100	1	1.95(1.04–3.66)	NA	NA	1	1.52(0.84–2.75)	NA	NA	2	1.71(1.11–2.63)	0.0	0.572
Gender												
male	3	0.981(0.50–1.30)	76.6	0.014	3	0.57(0.34–0.96)	77.6	0.012	2	0.09(0.002–5.43)	92.7	0.000
female	3	0.92(0.84–1.00)	50.7	0.072	3	0.81(0.63–1.03)	75.8	0.016	2	0.51(0.23–1.13)	86.2	0.000
